# Retraction: Relationship between rs1801157 polymorphism in stromal cell-derived factor gene and systemic lupus erythematosus risk

**DOI:** 10.18632/oncotarget.27351

**Published:** 2019-11-19

**Authors:** Can Qian, Qinghua Zou, Yong Wang

**Affiliations:** ^1^ Department of Rheumatology, Southwest Hospital, Third Military Medical University, Chongqing 400038, China

**This article has been retracted:** The authors recently reported analytical errors in this paper that drastically change the credibility of the published article conclusions. Major concerns include insufficient sample size, questionable literature search strategy, missing literature quality assessment and missing clinical heterogeneity analysis. All these concerns lead to the question of the reliability of its conclusion. Based on the problems identified in the assessment process and the results of institutional investigation, all authors have concluded that the most appropriate course of action is to retract the paper.

Original article: Oncotarget. 2017; 8:75509–75515. 75509-75515. https://doi.org/10.18632/oncotarget.19766

